# Supplementary figures and images for: Impairments in SHMT2 expression or cellular folate availability reduce oxidative phosphorylation and pyruvate kinase activity
Source: Genes Nutr. 2023 Mar 24;18:5. doi: 10.1186/s12263-023-00724-3 (PMC10037823; doi:10.1186/s12263-023-00724-3)

## Slide 1
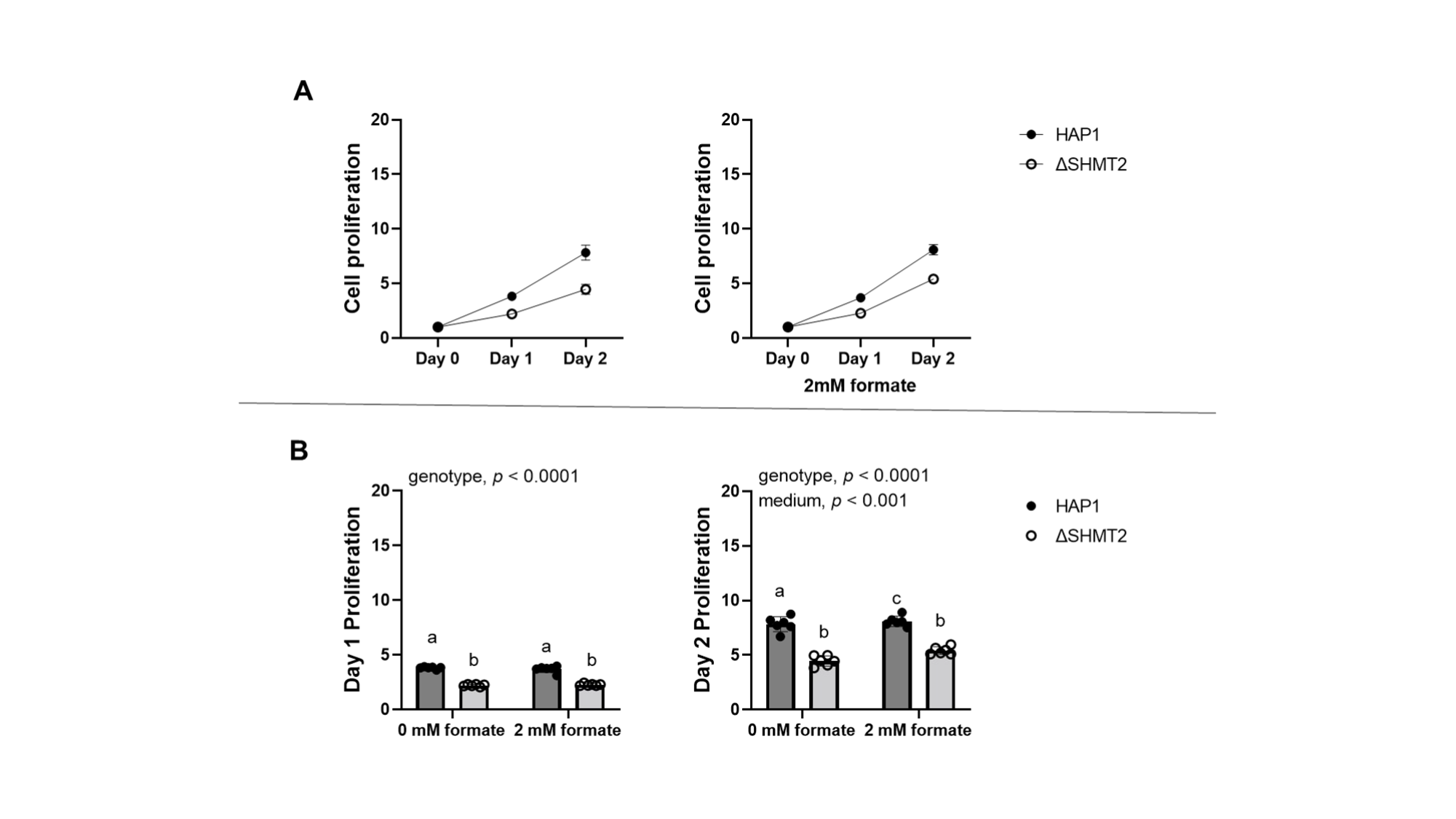

Supplement: Supplementary file 1 — Additional file 1: Figure S1. Cellular proliferation in HAP1 and ΔSHMT2 cells cultured in IMDM medium. Formate supplemented medium has no impact on rescuing the impaired cellular proliferation in ΔSHMT2 cells. Cell proliferation rates of ΔSHMT2 cells were compared with HAP1 cells by co-staining cells with Hoechst 33342 (to identify all cells) and propidium iodide (to identify dead cells). Fold change of each group was calculated by dividing by day 0 cell number. Data represent means ± SD values. Values represent n = 6 replicates of cell lines cultured in folate-sufficient IMDM medium. A) Cell proliferation rate and cell proliferation rate in the presence of 2 mM formate and B) relative day quantitation cell proliferation rate in the presence of 2 mM formate. Linear mixed-effects models with main effects of media, genotype, and time (with time as a continuous variable), and 2- and 3-way interactions were used to determine cell proliferation with a statistical significance at p < 0.05. Two-way ANOVA with Tukey’s post-hoc analysis was used to determine media by genotype interaction and main effects of media and genotype with a statistical significance at p < 0.05 were used to analyze individual day proliferation. Levels not connected by the same letter are significantly different. [file 12263_2023_724_MOESM1_ESM.pptx]
